# Preditores de Recorrência de Taquiarritmia Atrial após Ablação por Cateter: Impacto Socioeconômico em Países de Baixa e Média Renda

**DOI:** 10.36660/abc.20250248

**Published:** 2025-08-06

**Authors:** Marwan Shawki, Thalys Sampaio Rodrigues, Levindo Jose Garcia Quarto

**Affiliations:** 1 Austin Health Department of Cardiology Melbourne Victoria Austrália Department of Cardiology, Austin Health, Melbourne, Victoria – Austrália; 2 University of Melbourne Dentistry and Health Sciences Faculty of Medicine Melbourne Victoria Austrália Faculty of Medicine, Dentistry and Health Sciences, University of Melbourne, Melbourne, Victoria – Austrália; 3 St Elizabeth Hospital Department of Cardiology Boston MA EUA Department of Cardiology, St Elizabeth Hospital, Boston, MA – EUA

**Keywords:** Disparidades Socioeconômicas em Saúde, Ablação por Cateter, Fibrilação

**Ao Editor,**

Lemos com grande interesse o artigo de Ternes et al., recentemente publicado nos Arquivos Brasileiros de Cardiologia, juntamente com seu editorial elucidativo.^1 , 2^ Este estudo prospectivo, multicêntrico, do tipo coorte, realizado com pacientes consecutivos em três centros no Brasil, forneceu informações valiosas sobre os preditores de recorrência de arritmia atrial após primeira ablação por cateter. O estudo destacou o contexto clínico em um país de renda média e relatou desfechos semelhantes aos de países de alta renda. No entanto, os resultados devem ser interpretados com cautela, e alguns pontos merecem mais discussão.

Primeiramente, fatores socioeconômicos desempenham um papel importante no acesso dos pacientes à ablação por cateter e influenciam os desfechos gerais.^3 , 4^ Está bem documentado que, em comparação a países de alta renda, os pacientes com fibrilação atrial (FA) apresentam piores desfechos em países de baixa e média renda, em termos de mortalidade, anos de vida perdidos e anos de vida ajustados por incapacidade.^5^ Um dado interessante é que, mesmo em países de alta renda, parece haver diferença nos desfechos da ablação de FA com base nas faixas de renda.^6 , 7^ Acreditamos que os resultados atuais reflitam a prática privada do sul do Brasil, e que as inferências sobre os desfechos de um país de renda média devam ser interpretadas nesse contexto. Um maior esclarecimento sobre os caminhos de encaminhamento para esses centros privados bem como o perfil socioeconômico dos pacientes recrutados deve ser considerado para melhor contextualização dos resultados.

Em segundo lugar, no contexto de um acesso mais amplo dos pacientes, o intervalo entre o diagnóstico de FA e a ablação por cateter é uma variável fundamental que não foi suficientemente descrita. Por exemplo, na Austrália, um país de alta renda, possuir plano de saúde privado resultou em uma chance maior e mais rápida de realizar ablação por cateter em comparação a pacientes usuários do sistema público de saúde.^7^ Assim, obter mais informações sobre a condição socioeconômica e o status de saúde dos pacientes incluídos no estudo de Ternes et al., bem como sobre o tempo decorrido entre o diagnóstico da FA e a ablação, forneceria dados adicionais para comparação com países de alta renda.^1^

Além disso, os métodos utilizados para detectar a recorrência de taquicardia atrial, como a porcentagem de pacientes que realizaram monitoramento Holter após a ablação por cateter e a duração desse monitoramento, não foram suficientemente detalhados. Esses dados são cruciais para fornecer evidências de uma avaliação mais precisa da carga da taquiarritmia atrial.

Reconhecemos os desafios em oferecer ablação por cateter em tempo hábil para arritmias atriais, especialmente no Brasil, e parabenizamos os autores por este importante estudo. Para destacar tais disparidades, sugerimos que estudos futuros sejam conduzidos com populações do mundo real, provenientes de diferentes regiões do Brasil e com distintos perfis socioeconômicos.

## References

[1] Ternes CMP, Rohde LE, Forno AD, Lewandowski A, Nascimento HG, Odozynski G (2025). The Southern Brazilian Registry of Atrial Fibrillation (SBR-AF Registry): Predictors of Atrial Arrhythmia Recurrence after First-Time Catheter Ablation. Arq Bras Cardiol.

[2] Chokr MO (2025). Southern Brazil Registry of Atrial Fibrillation (SBR-AF): Predictors of Atrial Arrhythmia Recurrence after First Catheter Ablation. Arq Bras Cardiol.

[3] Eberly LA, Garg L, Yang L, Markman TM, Nathan AS, Eneanya ND (2021). Racial/Ethnic and Socioeconomic Disparities in Management of Incident Paroxysmal Atrial Fibrillation. JAMA Netw Open.

[4] Olsen F, Uleberg B, Jacobsen BK, Heuch I, Tande PM, Bugge E (2022). Socioeconomic and Geographic Differences in Ablation of Atrial Fibrillation in Norway - A National Cohort Study. BMC Public Health.

[5] Santos IS, Goulart AC, Olmos RD, Thomas GN, Lip GYH, Lotufo PA (2020). Atrial Fibrillation in Low- and Middle-Income Countries: A Narrative Review. Eur Heart J Suppl.

[6] Vinter N, Calvert P, Kronborg MB, Cosedis-Nielsen J, Gupta D, Ding WY (2023). Social Determinants of Health and Catheter Ablation after an Incident Diagnosis of Atrial Fibrillation: A Danish Nationwide Cohort Study. Eur Heart J Qual Care Clin Outcomes.

[7] Quiroz JC, Brieger D, Jorm LR, Sy RW, Falster MO, Gallego B (2022). An Observational Study of Clinical and Health System Factors Associated with Catheter Ablation and Early Ablation Treatment for Atrial Fibrillation in Australia. Heart Lung Circ.

