# Supplementary material for: Preditores de Recorrência de Taquiarritmia Atrial após Ablação por Cateter: Impacto Socioeconômico em Países de Baixa e Média Renda
Source: Arq Bras Cardiol. 2025 Aug 6;122(7):e20250248. [Article in Portuguese] doi: 10.36660/abc.20250248 (PMC12695035; doi:10.36660/abc.20250248)
Supplement: Translation

# Translation: Predictors of Atrial Arrhythmia Recurrence after Catheter Ablation: Socioeconomic Impact in Low to Middle-Income Countries

**DOI:** 10.36660/abc.20250248i

**Authors:** Marwan Shawki, Thalys Sampaio Rodrigues, Levindo Jose Garcia Quarto

**Affiliations:** 1 Melbourne Victoria Australia Department of Cardiology, Austin Health, Melbourne, Victoria – Australia; 2 Melbourne Victoria Australia Faculty of Medicine, Dentistry and Health Sciences, University of Melbourne, Melbourne, Victoria – Australia; 3 Boston MA USA Department of Cardiology, St Elizabeth Hospital, Boston, MA – USA

**Keywords:** Socioeconomic Disparities in Health, Catheter Ablation, Atrial Fibrillation

**To the Editor,**

We read with great interest the article by Ternes et al., recently published in the Arquivos Brasileiros de Cardiologia, with its accompanying insightful editorial.^1 , 2^This multicentre prospective cohort study of consecutive patients across three centres in Brazil provided valuable insights into the predictors of atrial arrhythmia recurrence following first-time catheter ablation. It highlighted its clinical setting in a middle-income country and reported outcomes similar to those in high-income countries. However, the results must be interpreted cautiously, and some points merit further discussion.

Firstly, socioeconomic factors play an important role in patient accessibility to catheter ablation and affect overall outcomes.^3 , 4^It is well described that, compared to high-income countries, patients with atrial fibrillation (AF) have poorer outcomes in low-middle-income countries in terms of death, years of life lost and disability-adjusted life years.^5^Interestingly, even in high-income countries, there appears to be a difference in AF ablation outcomes based on income brackets.^6 , 7^We believe that current results reflect Southern Brazil's private practice and inferences about the outcomes of a middle-income country should be interpreted in that context. Further clarification of the referral pathways to these private centres and the socioeconomic status of recruited patients should be considered to better contextualise results.

Secondly, in the context of broader patient accessibility, the time from the diagnosis of AF to catheter ablation is a pivotal variable that was not sufficiently described. For instance, in Australia, a high-income country, holding private healthcare insurance resulted in a quicker and higher chance of receiving a catheter ablation than publicly covered patients.^7^Hence, having more insights about the socioeconomic and healthcare status of patients included in the study by Ternes and colleagues, and the subsequent interval from AF diagnosis to catheter ablation, would provide further information regarding benchmarking against high-income countries.^1^

Furthermore, the methods employed to detect the recurrence of atrial tachycardia, such as the percentage of patients who received Holter monitoring post-catheter ablation and the duration of monitoring, were not sufficiently detailed. This is crucial for providing evidence of a more accurate assessment of the burden of atrial tachyarrhythmia.

We appreciate the challenges in providing timely catheter ablation for atrial arrhythmias, especially in Brazil, and commend the authors for this important study. To highlight such disparities, we suggest that further studies should be carried out to include real-world populations from various regions across Brazil and with different socioeconomic backgrounds.
