# Supplementary material for: Preditores de Recorrência de Taquiarritmia Atrial após Ablação por Cateter: Impacto Socioeconômico em Países de Baixa e Média Renda
Source: Arq Bras Cardiol. 2025 Aug 6;122(7):e20250248. [Article in Portuguese] doi: 10.36660/abc.20250248 (PMC12695035; doi:10.36660/abc.20250248)
Supplement: Related texts

# Reply: Carta-resposta

**Authors:** Caique M. P. Ternes, Andre d’Avila

**Affiliations:** 1 Hospital SOS Cárdio Serviço de Arritmia Cardíaca Florianópolis SC Brasil Serviço de Arritmia Cardíaca, Hospital SOS Cárdio, Florianópolis, SC – Brasil; 2 Baylor College of Medicine Cardiovascular Research Institute Houston Texas EUA Cardiovascular Research Institute, Baylor College of Medicine, Houston, Texas – EUA; 3 Harvard-Thorndike Electrophysiology Institute Beth Israel Deaconess Medical Center Harvard Medical School Boston Massachusetts EUA Harvard-Thorndike Electrophysiology Institute, Beth Israel Deaconess Medical Center, Harvard Medical School, Boston, Massachusetts – EUA

Agradecemos os comentários pertinentes apresentados na Carta ao Editor sobre nosso artigo, "Registro SBR-AF: Preditores de Recorrência de Taquiarritmia Atrial após Primeira Ablação por Cateter na Fibrilação Atrial".^1^Os pontos levantados sobre fatores socioeconômicos, vias de encaminhamento e métodos de monitoramento são valiosos e merecem esclarecimentos adicionais para contextualizar nossos achados.

Nossa coorte de estudo, composta por 1043 pacientes consecutivos, foi, de fato, atendida em três instituições privadas na região Sul do Brasil. Vale destacar que Santa Catarina contribuiu com quase 600 das aproximadamente 6000 ablações de Fibrilação Atrial (FA) realizadas no Brasil em 2024, representando 10% do total nacional, apesar de abrigar apenas 3,8% da população brasileira, com cerca de 8 milhões de habitantes.^2^Isso destaca a significativa contribuição da região aos procedimentos de ablação de FA, provavelmente impulsionada por sua robusta infraestrutura de saúde e indicadores socioeconômicos mais elevados em comparação a outros estados brasileiros. Por exemplo, Santa Catarina possui uma renda *per capita mensal* de aproximadamente R$ 2.200, significativamente superior à média nacional de R$ 1.625.^2^Além disso, seu Índice de Desenvolvimento Humano (IDH), de 0,792, está entre os mais altos do Brasil. Esses aspectos socioeconômicos provavelmente facilitam o acesso a cuidados especializados, incluindo ablação por cateter, e podem contribuir para melhores desfechos em saúde.

Reconhecemos que fatores socioeconômicos podem impactar o tratamento e o controle de comorbidades como hipertensão, diabetes e obesidade, as quais são conhecidamente associadas ao aumento do risco de recorrência de FA após ablação.^3^No entanto, nossos achados sugerem que um procedimento de ablação tecnicamente adequado pode mitigar alguns desses riscos. Por exemplo, nosso estudo relatou uma baixa taxa de complicações de 2,1% e taxas de recorrência comparáveis às de países de alta renda (19% para FA paroxística e 30% para FA persistente após o primeiro procedimento), destacando o potencial da assistência intervencionista de alta qualidade para superar certas limitações socioeconômicas.

Concordamos que a ausência de dados detalhados sobre o tempo entre o diagnóstico da FA e a ablação limita nossa capacidade de realizar comparações mais precisas com países de alta renda. Da mesma forma, concordamos que os métodos utilizados para detectar recorrências assintomáticas de FA – como o uso de monitoramento implantável para definir a carga arrítmica, em vez de uma definição binária simplista de recorrência – representa uma limitação importante. Sem um sistema de monitoramento mais robusto, as recorrências assintomáticas podem ser subnotificadas, subestimando potencialmente a real carga das taquiarritmias atriais. Infelizmente, essa é uma limitação relevante do nosso registro prospectivo. Estudos futuros devem incorporar protocolos de monitoramento padronizados de longo prazo para lidar com essa limitação.

Para melhor abordar as disparidades, certamente apoiamos a iniciativa de realização de estudos que abranjam diferentes regiões do Brasil e diversos perfis socioeconômicos. Expandir a pesquisa para outros centros, incluindo o sistema público de saúde e populações sub-representadas, proporcionaria uma compreensão mais abrangente dos desfechos da ablação de FA em todo o país. Agradecemos aos autores pela crítica construtiva e esperamos que nosso estudo sirva de base para futuras pesquisas direcionadas ao acesso amplo e eficaz à assistência no tratamento da FA em países de baixa e média renda

**Atenciosamente,**

**Caique Ternes**

**Andre d’Avila**

## References

[1] Ternes CMP, Rohde LE, Forno AD, Lewandowski A, Nascimento HG, Odozynski G (2025). The Southern Brazilian Registry of Atrial Fibrillation (SBR-AF Registry): Predictors of Atrial Arrhythmia Recurrence after First-Time Catheter Ablation. Arq Bras Cardiol.

[2] Instituto Brasileiro de Geografia e Estatística (2025). [Internet].

[3] Santos IS, Goulart AC, Olmos RD, Thomas GN, Lip GYH, Lotufo PA (2020). Eur Heart J Suppl.

# Reply

**Authors:** Caique M. P. Ternes, Andre d’Avila

**Affiliations:** 1 Florianópolis SC Brazil Serviço de Arritmia Cardíaca, Hospital SOS Cárdio, Florianópolis, SC – Brazil; 2 Houston Texas USA Cardiovascular Research Institute, Baylor College of Medicine, Houston, Texas – USA; 3 Boston Massachusetts USA Harvard-Thorndike Electrophysiology Institute, Beth Israel Deaconess Medical Center, Harvard Medical School, Boston, Massachusetts – USA

We appreciate the thoughtful comments provided in the Letter to the Editor regarding our article, "The Southern Brazilian Registry of Atrial Fibrillation (SBR-AF Registry): Predictors of Atrial Arrhythmia Recurrence after First-Time Catheter Ablation".^1^The points raised about socioeconomic factors, referral pathways, and monitoring methods are valuable and warrant further clarification to contextualize our findings.

Our study cohort of 1,043 consecutive patients was indeed treated across three private institutions in Southern Brazil. Notably, Santa Catarina accounted for nearly 600 of the estimated 6,000 atrial fibrillation (AF) ablations performed in Brazil in 2024, representing 10% of the national total despite comprising only 3.8% of Brazil's population, with approximately 8 million inhabitants.^2^This highlights the region's significant contribution to AF ablation procedures, likely driven by its robust healthcare infrastructure and higher socioeconomic indicators compared to other Brazilian states. For instance, Santa Catarina boasts a *per capita* income of approximately BRL 2,200 per month, significantly higher than the national average of BRL 1,625.^2^Additionally, its Human Development Index (HDI) of 0.792 is among the highest in Brazil. These socioeconomic aspects likely facilitate better access to specialized care, including catheter ablation, and may contribute to improved health outcomes.

We acknowledge that socioeconomic factors can impact treatment and control of comorbidities such as hypertension, diabetes, and obesity, which are known to increase the risk of AF recurrence after ablation.^3^However, our findings suggest that a technically adequate ablation procedure can mitigate some of these risks. For example, our study reported a low complication rate of 2.1% and recurrence rates comparable to high-income countries (19% for paroxysmal AF and 30% for persistent AF after a first procedure), underscoring the potential of high-quality procedural care to overcome certain socioeconomic limitations.

We agree with that the lack of detailed data on the time from AF diagnosis to ablation limits our ability to fully benchmark against high-income countries. Similarly, we concur that the methods for detecting asymptomatic AF recurrence, such as implantable monitoring to define AF burden rather than a simplistic binary definition of recurrence imposes an important limitation. Without a more robust monitoring system, asymptomatic recurrences may be underreported, potentially underestimating the true burden of atrial tachyarrhythmia. Unfortunately, this is an important limitation of our prospective registry. Future studies should incorporate standardized, long-term monitoring protocols to address this limitation.

To further address disparities, we would certainly support the initiative for studies encompassing diverse regions of Brazil and varying socioeconomic backgrounds. Expanding research to other centers to include public healthcare settings and underrepresented populations would provide a more comprehensive understanding of AF ablation outcomes across Brazil. We thank the authors for their insightful critique and hope our study serves as a foundation for future investigations into equitable access to AF care in low- and middle-income countries.

**Sincerely,**

**Caique Ternes**

**Andre d’Avila**
